# Effect of acute hypobaric hypoxia on the endothelial glycocalyx and digital reactive hyperemia in humans

**DOI:** 10.3389/fphys.2014.00459

**Published:** 2014-11-24

**Authors:** Pär I. Johansson, Anita Bergström, Niels J. Aachmann-Andersen, Martin A. S. Meyer, Sisse R. Ostrowski, Nikolai B. Nordsborg, Niels V. Olsen

**Affiliations:** ^1^Section for Transfusion Medicine, Capital Region Blood Bank, The Diagnostic Centre, Copenhagen University Hospital (Rigshospitalet)Copenhagen, Denmark; ^2^Department of Surgery, University of Texas Medical SchoolHouston, TX, USA; ^3^Department of Neuroanaesthesia, The Neuroscience Centre, Copenhagen University Hospital (Rigshospitalet)Copenhagen, Denmark; ^4^BrainLab, Department of Neuroscience and Pharmacology, University of CopenhagenCopenhagen, Denmark; ^5^Department of Nutrition, Exercise and Sport, University of CopenhagenCopenhagen, Denmark

**Keywords:** endothelium, hypoxia, glycocalyx, nitrite/nitrate, peripheral arterial tonometry, protein C, syndecan-1

## Abstract

**Introduction:** Hypoxia is associated with increased capillary permeability. This study tested whether acute hypobaric hypoxia involves degradation of the endothelial glycocalyx.

**Methods:** We exposed 12 subjects to acute hypobaric hypoxia (equivalent to 4500 m for 2–4 h) and measured venous blood concentrations of biomarkers reflecting endothelial and glycocalyx degradation (catecholamines, syndecan-1, soluble CD40 ligand, protein C, soluble thrombomodulin, tissue-type plasminogen activators, histone-complexed DNA fragments, and nitrite/nitrate). Endothelial function was assessed by the hyperemic response to brachial artery occlusion by peripheral arterial tonometry.

**Results:** Compared with normoxic baseline levels, hypoxia increased concentrations of syndecan-1 from 22 (95% confidence interval: 17–27) to 25 (19–30) ng/ml (*p* < 0.02) and protein C from 76 (70–83)% to 81 (74–88)% (*p* < 0.02). Nitrite/nitrate decreased from 23 (18–27) μM at baseline to 19 (14–24) μM and 18 (14–21) μM in hypoxia and recovery, respectively (*p* < 0.05). Other biomarkers remained unchanged. The post-occlusion/pre-occlusion ratio (reactive hyperemia index, RHI) decreased from 1.80 (1.52–2.07) in normoxia to 1.62 (1.28–1.96) after 2–4 h of hypobaric hypoxia and thereafter increased to 2.43 (1.99–2.86) during normoxic recovery (*p* < 0.01).

**Conclusions:** The increase in syndecan-1 and protein C suggests that acute hypobaric hypoxia produces a minor degree of glycocalyx degradation and overall cellular damage. After hypoxia RHI rebounded to higher than baseline levels suggesting improved endothelial functionality.

## Introduction

Acute hypoxia rapidly activates endothelial cells leading to increased vascular permeability and initiation of prothrombotic and inflammatory reactions (Ten and Pinsky, 2002; Gonzalez and Wood, 2010). Hypoxic exposure may also result in endothelium-dependent vasoconstriction, both by release of endothelial-derived substances including endothelins, superoxide anions and thromboxane A_2_, and by a decrease in nitric oxide bioavailability (Schneider et al., 2001; Ten and Pinsky, 2002; Bärtsch et al., 2005; Gonzalez and Wood, 2010). In subjects susceptible to high altitude pulmonary edema (HAPE), strong evidence exists to suggest that impairment of systemic endothelial function is a main cause of enhanced hypoxic pulmonary vasoconstriction in these subjects (Busch et al., 2001; Swenson et al., 2002; Berger et al., 2005).

The luminal side of the endothelium is lined by the glycocalyx consisting of a 0.2–1 μm thick negatively charged, antiadhesive and anticoagulant carbohydrate-rich layer which protects the endothelium and contributes to its barrier function (Pries et al., 2000; Johansson et al., 2011). Degradation of the glycocalyx results in local thrombin formation, fibrinolysis, and adhesion of platelets and leucocytes (Vink et al., 2000; Nieuwdorp et al., 2006; Chappell et al., 2010). In trauma patients and patients with acute myocardial infarction, high levels of circulating syndecan-1, a transmembrane endothelial proteoglycan and a marker of endothelial glycocalyx degradation (Pries et al., 2000), is associated with inflammation, coagulopathy, and increased mortality (Johansson et al., 2011; Ostrowski et al., 2013a,b). Acute exposure to hypoxia may occur in high altitude and aviation activities and also acute oxygen deprivation encompasses several clinical modalities such as smoke intoxication, respiratory distress syndrome, pulmonary and cardiac thromboembolism, sepsis, and trauma. In this study, we evaluated whether exposure to short-term, acute hypobaric hypoxia is associated with degradation of the endothelial glycocalyx. The effect of short-term hypoxia *per se* was studied in healthy subject by the use of a low-pressure chamber. The response of endothelial cells to shear stress was assessed by digital pulse amplitude tonometry (Faizi et al., 2009; Hamburg and Benjamin, 2009; Hedetoft and Olsen, 2014).

## Materials and methods

### Subjects and experimental protocol

Twelve healthy males aged 25 (20–29) years (mean and range), height 181 (173–189) cm and body mass index 22 (18–26) kg/m^2^ entered the study after having given their written, informed consent. The study was approved by the Regional Ethical Committee of the Copenhagen Region, 2 Kongens Vaenge, DK-3400 Hillerød, Denmark, E-mail: regionh@regionh.dk (J.No. H-4-2011-080). All subjects were non-smokers living at sea level and free of disease and medication. After an overnight fast, the subjects arrived to the laboratory at 08.00 a.m. The subjects abstained from heavy physical exercise and alcohol intake in the preceding 24 h. Drinking water was provided freely during the experiment. The experiment was conducted inside a low-pressure chamber with four subjects in each session. After insertion of an intravenous catheter in a cubital vein, the subjects rested for at least 1 h in a sitting position that was maintained throughout the study period. Thereafter, baseline measurements by digital pulse amplitude tonometry and blood samples were obtained in normobaric normoxia (open chamber). The chamber was then decompressed (over 15–20 min) to a simulated altitude af 4500 m above sea level. This decompression produces a hypobaric hypoxia comparable to that obtained in high altitude laboratories (Pikes Peak, Colarado, USA; Regina Margherita Hut, Monte Rosa, Italy; and l'Observatoire Vallot, Mont Blanc, France). In each session, measurements in the four subjects, in succession one by one, were conducted within 2–4 h in hypobaric hypoxia. Finally, measurements were repeated in the recovery period 1–3 h after re-compression to ambient normoxic conditions. At the same time points heart rate and arterial pressure were measured and arterial blood was sampled for analysis of SaO_2_,PaCO_2_,PaO_2_, and pH, and concentrations of hemoglobin, glucose, and lactate by the use of a Radiometer ABL 725 device (Radiometer Medical A/S, Copenhagen, Denmark).

### Enzyme linked immunosorbent assay (ELISA) measurements

Soluble biomarkers of sympathoadrenal activation, endothelial cell and glycocalyx activation and damage, natural anticoagulation, fibrinolysis and platelet activation were measured at baseline, and again at recovery by commercially available immunoassays in plasma/serum according to the manufactures recommendations. *Adrenaline and noradrenaline* (sympathoadrenal activation) were measured in EDTA plasma by a 2-CAT ELISA, Labor Diagnostica Nord GmbH & Co. KG, Nordhorn, Germany. Lower limits of detection (LLD) were 10 pg/ml (normal reference <100 pg/ml) and 50 pg/ml (normal reference <600 pg/ml), respectively. *Histone-complexed DNA fragments* (endothelial cell damage) in EDTA plasma were measured by a Cell Death Detection ELISAPLUS, Roche, Hvidovre, Denmark (LLD not stated, relative quantification). *Soluble CD40 ligand* (pro-inflammatory activation) was determined by ELISA (sCD40L, R&D Systems Europe; LLD 4.2 pg/ml). *Soluble thrombomodulin* (natural anticoagulation) and *Protein C* (endothelial cell damage) were measured in citrate plasma (sTM, Nordic Biosite, Copenhagen, Denmark, LLD 0.38 ng/ml; and PC, Helena Laboratories, Beaumont, TX, US, LLD not stated, relative quantification). *Tissue-type plasminogen activators* (fibrinolysis and platelet activation) were measured in citrate plasma (tPA, ADI, detects sc-tPA, tc-tPA, and tPA/PAI-1 complexes; LLD 1 ng/ml). *Syndecan-1* (glycocalyx activation and damage) was determined in serum (Diaclone SAS, Besancon, France; LLD 4.94 ng/ml). Samples for measurement of *nitrite/nitrate* were spun through a 30-kD micropore filter (Nanosep 30k Omega, Pall Corp., Ann Arbor, Michigan) prior to duplicate analysis with a commercially available NOx detection kit based on the Griess reaction (cat. 780001, Nitrite/Nitrate Colorimetric Assay Kit, Cayman Chemicals, Ann Arbor, Michigan).

### Digital pulse amplitude tonometry

We used an EndoPAT 2000 device (Itamar Medical Ltd., Caesarea, Israel) consisting of a fingertip plethysmograph (Faizi et al., 2009; Hamburg and Benjamin, 2009; Hedetoft and Olsen, 2014). The device includes two fingerprobes, each placed on a fingertip on each hand. These are used for parallel measurements and are connected to a computer. The probe consists of a rigid external cap around an air-filled chamber with a sensor. When the chamber is filled with air, a uniform pressure is provided which prevents the veno-arteriolar vasoconstrictive reflex. The probe detects changes in volume in relation to the arterial pulsation. A cuff was placed on the arm in which the measurement was performed. Measurements by the other probe served as a control. Each measurement consisted of three phases: baseline, occlusion and reactive hyperemia. For baseline measurements, the probe was set to inflate to 10 mmHg below diastolic pressure. For occlusion, the test arm was occluded to suprasystolic pressure for 5 min (Faizi et al., 2009). The subsequent increase in blood flow leads to a flow-mediated dilatation, manifesting as reactive hyperemia, which was measured by the device as an increase in the pulse-signal amplitude. The EndoPAT software calculates a post-occlusion/pre-occlusions-ratio, the reactive hyperemia index (RHI). An RHI ≤ 1.67 is described as being abnormal by the manufacturer (http://www.itamar-medical.com/images/EndoPAT Multi Function USA.pdf).

### Statistical analysis

Statistical analysis was performed using IBM SPSS Statistics 20 (IBM SPSS Statistics, Armonk, NY). Values during hypoxia and in the post-hypoxic recovery period were compared with baseline by the use of paired *t*-tests. Correlations between EndoPAT RHI values and endothelial markers that had changed significantly during the experiment, and between RHI and NOx values, all at identical time points, were investigated by Pearson's and Spearman's correlation as well as linear and logarithmic regression (by curve estimation). A level of *P* < 0.05 was considered statistical significant. Results are presented as means with 95% confidence intervals.

## Results

Heart rate and mean arterial blood pressure remained unchanged (Table 1). PaO_2_, PaCO_2_ and O_2_ saturation decreased during hypoxia, whereas pH and lactate increased (Table 1); values were restored to baseline levels during the ensuing period of normoxia (recovery) with the exception of PaCO_2_ that remained lower than baseline levels (Table 1). Also plasma hemoglobin concentration at recovery was lower compared with baseline (Table 1). Venous plasma concentrations of nitrite/nitrate decreased during hypoxia compared with baseline, and remained at a similar level during recovery (Table 1).

**Table 1:** **Effect of hypobaric hypoxia**.

	**Baseline**	**Hypoxia**	**Recovery**
Heart rate (min^−1^)	70 (62–77)	75 (69–81)	68 (62–74)
MABP (mm Hg)	90 (85–95)	83 (76–90)	84 (77–90)
pH	7.40 (7.39–7.41)	7.47 (7.46–7.49)^*^	7.42 (7.39–7.45)
P_a_O_2_ (mm Hg)	103 (99–107)	43 (39–46)^*^	102 (98–106)
P_a_CO_2_ (mm Hg)	40 (38–42)	32 (30–35)^*^	36 (34–37)^*^
S_a_O_2_ (%)	98 (98–98)	81 (77–85)^*^	99 (98–99)
Hgb (mM)	9.8 (9.1–10.4)	9.3 (9.0–9.6)	9.2 (8.8–9.5)^*^
Lactate (mM)	0.84 (0.65–1.02)	1.29 (1.05–1.53)^*^	0.90 (0.63–1.17)
NOx (μM)	22.7 (18.3–27.1)	19.2 (14.4–24.1)	17.8 (14.6–21.1)^*^

*Data are presented as means with 95% confidence intervals. n = 12, exept for NOx where n = 11 in hypoxia and recovery. MABP, mean arterial blood pressure; P_a_O_2_, arterial oxygen tension; P_a_CO_2_, arterial CO_2_ tension; S_a_O_2_, arterial oxygen saturation; Hgb, hemoglobin concentration; NOx, nitrite/nitrate concentration*.

* *p < 0.05 compared with baseline*.

Syndecan-1 and protein C increased from the baseline levels to the recovery levels (Table 2). Concentrations of adrenaline, noradrenaline, thrombomodulin, tissue-type plasminogen activators, plasminogen activator inhibitor 1, soluble CD40 ligand, and histone-complexed DNA fragments remained unchanged (Table 2).

**Table 2:** **Venous concentrations of catecholamines and endothelial markers before and after hypobaric hypoxia**.

	**Baseline**	**Recovery**
Adrenaline (pg/ml)	84 (38–129)	57 (31–82)
Noradrenaline (pg/ml)	199 (117–280)	234 (134–334)
Syndecan-1 (ng/ml)	22 (17–27)	25 (19–30)^*^
PC (%)	76 (70–83)	81 (74–88)^*^
sTM (ng/ml)	6.0 (5.2–6.8)	6.1 (5.3–6.9)
tPA (ng/ml)	3.1 (1.5–4.7)	3.3 (1.4–5.3)
PAI-1 (ng/ml)	5.7 (0.3–11.2)	5.5 (-0.6–11.6)
sCD40L (pg/ml)	41 (13–68)	33 (24–42)
hcDNA (%)	2.6 (2.0–3.2)	2.6 (2.2–2.9)

*Data are presented as means with 95% confidence limits. n = 11. PC, protein C; sTM, soluble thrombomodulin; tPA, tissue plasminogen activators; PAI-1, plasminogen activator inhibitor 1; sCD40L, soluble CD40 ligand; hcDNA, histone-complexed DNA fragments*.

* *p < 0.02 compared with baseline*.

**Figure 1:**
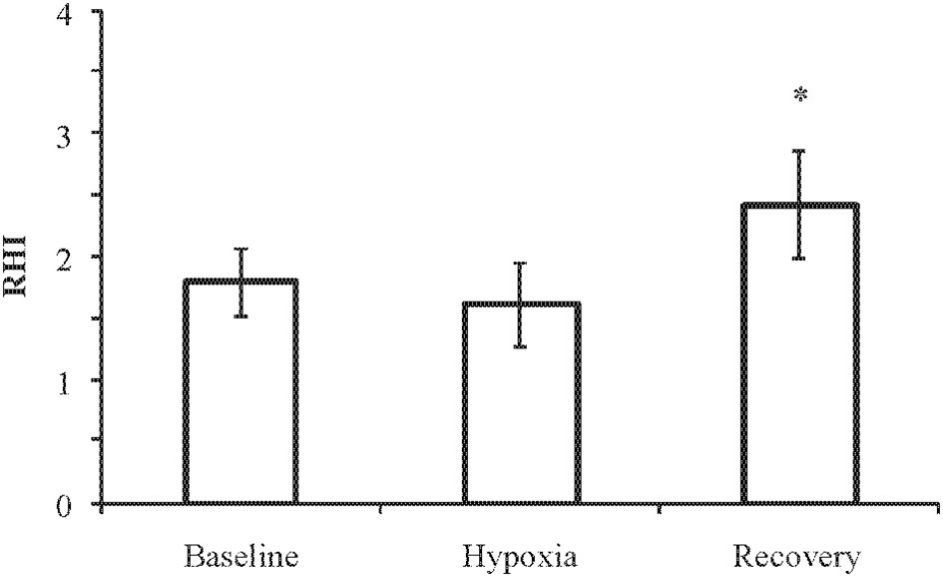
**EndoPAT reactive hyperemia index (RHI) at baseline (*n* = 11), hypoxia (4500 m for 2–4 h, *n* = 7), and recovery (*n* = 12)**. Data are presented as means with 95% confidence intervals. ^*^p = 0.008 compared with baseline.

Measurements with the EndoPAT device could not be obtained in one subject during baseline. Unexpected, the fingertip plethysmograph of the EndoPAT device failed to produce readable tonometry (pulse-signal amplitude) in 5/12 subjects during hypoxia. These subjects were the most affected by the decompression and presented with peripheral vasoconstriction. EndoPAT RHI fell from 1.80 (1.52–2.07, *n* = 11) at baseline to 1.62 (1.28–1.96) during hypoxia (*n* = 7; *p* = 0.11) but in the period of post-hypoxia increased significantly to 2.43 (1.99–2.86, *n* = 12) (Figure 1). At baseline 5/11 of the subjects had a RHI below 1.67 which is denoted as abnormal by the manufacturer of the EndoPAT device. During hypoxia 4/7 of the subjects had RHI values below 1.67; after hypoxia only 1/12 had a RHI below 1.67. RHI values at baseline and recovery did not correlate with corresponding values of syndecan-1, protein C, or nitrite/nitrate. Nor did RHI during hypoxia correlate with nitrite/nitrate values during hypoxia. Nitrite/nitrate did not correlate with neither syndecan-1 or protein C. No trend was observed regarding degree of degradation of endothelial glycocalyx and reactive hyperemia in relation to exposure time to hypobaric conditions.

## Discussion

The current study suggests that acute, short-term hypobaric hypoxia in humans is associated with minor degradation of the endothelium glycocalyx as reflected by the small increase in syndecan-1. Increased plasma concentrations of protein C indicate that acute hypobaric hypoxia induced some degree of cellular damage. At the same time, a decrease in venous concentration of nitrite/nitrate was observed. RHI after hypoxic exposure rebounded to higher than baseline levels. The present results are in line with previous studies in isolated, perfused hearts of guinea pigs (Annecke et al., 2011; Becker et al., 2011), and of rats (Ward and Donelly, 1993) showing that hypoxia initiates degradation of the coronary endothelial glycocalyx.

Acute hypoxia has often been shown to impair endothelial function. Earlier studies showed an increased transcapillary escape rate of albumin after 3.5 h and 2 days at 4500 m (Parving, 1972; Hansen et al., 1994). Berger et al. (2005) have shown that the endothelium dependent vasodilator responsiveness of forearm resistance vessels was blunted by acute normobaric hypoxia in subjects susceptible to HAPE but not in HAPE resistant subjects. Exhaled nitric oxide and pulmonary nitric oxide bioavailability is reduced in HAPE susceptible subjects (Busch et al., 2001; Swenson et al., 2002; Bailey et al., 2010). The present hypoxia induced decrease in systemic venous concentration of nitrite/nitrate is in accordance with previous studies (Bailey et al., 2010; Beall et al., 2012). However, this is associated with an increase in arterial nitrosoproteins and nitric oxide in red blood cells (Bailey et al., 2010). The nitric oxide pathways in hypoxia may both involve down-regulation of endothelial nitric oxide synthase (Beall et al., 2012), and up-regulation of nitric oxide formation from nitrite (Lundberg et al., 2008; David Ho et al., 2012), but exactly how these interactions are related to the RHI signal remains unknown. Interestingly, although hypoxia tended to reduce RHI, it increased above baseline levels in the post-hypoxia period, suggesting a beneficial role of hypoxia and its reversal on endothelial integrity. It could be speculated that this response, in part, may be involved in the beneficial effect of hypoxic “conditioning” (Przyklenk, 2013; Thielmann et al., 2013).

Increased levels of catecholamines, syndecan-1 and soluble thrombomodulin in patients with severe trauma, acute myocardial infarction and sepsis are associated with increased mortality (Johansson et al., 2011, 2012, 2013; Ostrowski et al., 2013a,b). In the present study, short-term hypobaric hypoxia did not induce significant increases in circulating catecholamines indicating that an effect on the endothelium from symphathetic outflow was negligible. In agreement with this, only modest increases in syndecan-1 was observed and, furthermore, no change in soluble thrombomodulin levels was found, indicating that no major damage on the endothelial cells was inflicted. We have previously reported that experimental endotoxemia in healthy volunteers receiving a 4-h 0.5 ng/kg/h infusion of *E. coli* lipopolysaccharide did not result in significant changes of circulating levels of catecholamines, syndecan-1 or soluble thrombomodulin contrary to patients with sepsis (Ostrowski et al., 2013a). Together with the RHI results, this suggests that the hypoxia model used in the present study resulted in a stronger endothelial impact than the LPS model, albeit much weaker than in patients with sepsis. The current level of hypoxia with an arterial oxygen saturation around 80% is often observed following poisoning with cyanide or carbonmonoxide in smoke inhalation injuries, and in the acute course of several clinical syndromes, i.e., sepsis, trauma, pulmonary thromboembolism and myocardial infarction. We found increased levels of syndecan-1 indicating shedding of the glycocalyx which has been associated with poor outcome in trauma patients (Johansson et al., 2011, 2012), whereas RHI increased as a measure of improved endothelial functionality. The significance of these findings requires further investigation.

We used a non-invasive EndoPat 2000 device for evaluation of peripheral endothelial function by peripheral arterial tonometry (PAT) (Hamburg and Benjamin, 2009; Patvardhan et al., 2010; Hedetoft and Olsen, 2014). The method is based on amplitude tonometry in the finger tip and provides easy and standardized measurements of a RHI value following a 5-min upper arm occlusion. A study in healthy individuals indicated that RHI, in part, depends on flow-mediated release of nitric oxide (Nohria et al., 2006). RHI is decreased in the presence of cardiovascular risk factors (Bonetti et al., 2004; Hamburg et al., 2008, 2011), and measurements of RHI may be used to predict risk of adverse cardiovascular events after surgery (Gokce et al., 2002) and acute coronary syndrome (Rubinshtein et al., 2010). Although patterns of abnormalities measured by PAT follow the same trend as that measured by brachial artery ultrasound in response to flow-mediated vasodilatation (Kuvin et al., 2003), it has become clear that the methods are not interchangeable (Lind et al., 2005; Dhingsa et al., 2008; Hamburg et al., 2011). Endothelial function in the brachial conduit artery and the smaller resistance vessels in the finger are governed by different mechanisms, and the two methods provide distinct information about endothelial function (Lind et al., 2005; Dhingsa et al., 2008; Hamburg et al., 2011). Nonetheless, the use of PAT may provide new ways to study the interaction between hypoxia and peripheral flow dynamics.

## Limitations

The time course in hypoxia plays an important role. Whereas short-term hypoxia may result in vasoconstriction in pulmonary and renal vessels and vasodilation in skeletal muscle, alterations in glycocalyx may require longer time (Pries et al., 2000; Vink et al., 2000; Nieuwdorp et al., 2006; Chappell et al., 2010; Johansson et al., 2011; Ostrowski et al., 2013a,b). Thus, the present use of only 2–4 h of hypoxia may have been too short to reveal the potential effect on the glycocalyx. In addition, the failure of obtaining fingertip plethysmographic signals with the EndoPAT device during hypoxia unfortunately weakened the study. Thus, the study would have benefited from inclusion of other measurements like flow-mediated vasodilatation in the brachial artery and nitric oxide bioavailability in red blood cells and exhaled air. Moreover, assessment of biochemical markers of glycocalyx degradation are indirect methods that are not comparable to direct investigation of the glycocalyx by electron-microscopy or confocal microscopy.

## Concluding remarks

The increase in syndecan-1 and protein C suggests that acute hypobaric hypoxia produces minor degree of glycocalyx degradation and overall cellular damage. After hypoxia RHI rebounded to higher than baseline levels suggesting improved endothelial functionality.

## Author contributions

Pär I. JohanssonĆ Anita Bergström and Niels J. Aachmann-Andersen: Principal contribution to design of the work acquisition analysis and interpretation of data. Principal drafting revising and (final) approval of the manuscript Accountable for all aspects of the work. Martin A. S. Meyer, Sisse R. Ostrowski and Nikolai B. Nordsborg: Important contribution to design of the work, analysis, and interpretation of data. Contributed to drafting, revising, and (final) approval of the manuscript. Accountable for all aspects of the work. Niels V. Olsen: Principal contribution to design of the work, analysis, and (final) approval of the manuscript. Accountable for all aspects of the work.

### Conflict of interest statement

The authors declare that the research was conducted in the absence of any commercial or financial relationships that could be construed as a potential conflict of interest.
